# Osthole Enhances Osteogenesis in Osteoblasts by Elevating Transcription Factor Osterix via cAMP/CREB Signaling In Vitro and In Vivo

**DOI:** 10.3390/nu9060588

**Published:** 2017-06-08

**Authors:** Zhong-Rong Zhang, Wing Nang Leung, Gang Li, Siu Kai Kong, Xiong Lu, Yin Mei Wong, Chun Wai Chan

**Affiliations:** 1 School of Chinese Medicine, Faculty of Medicine, The Chinese University of Hong Kong, Shatin, Hong Kong, China; zhang_zhongrong@cuhk.edu.hk (Z.-R.Z.); awnleung@gmail.com (W.N.L.); wongyinmei@cuhk.edu.hk (Y.M.W.); 2 Department of Orthopaedics and Traumatology, Faculty of Medicine, The Chinese University of Hong Kong, Shatin, Hong Kong, China; gangli@cuhk.edu.hk; 3 School of Life Sciences, The Chinese University of Hong Kong, Shatin, Hong Kong, China; skkong@cuhk.edu.hk; 4 Key Lab of Advanced Technologies of Materials, Ministry of Education, School of Materials Science and Engineering, Southwest Jiaotong University, Chengdu 610031, China; luxiong@home.swjtu.edu.cn

**Keywords:** osthole, osteoblast, osteogenesis, bone regeneration, fracture repair, cAMP/CREB signaling

## Abstract

Anabolic anti-osteoporotic agents are desirable for treatment and prevention of osteoporosis and fragility fractures. Osthole is a coumarin derivative extracted from the medicinal herbs *Cnidium monnieri* (L.) Cusson and *Angelica pubescens* Maxim.f. Osthole has been reported with osteogenic and anti-osteoporotic properties, whereas the underlying mechanism of its benefit still remains unclear. The objective of the present study was to investigate the osteopromotive action of osthole on mouse osteoblastic MC3T3-E1 cells and on mouse femoral fracture repair, and to explore the interaction between osthole-induced osteopromotive effect and cyclic adenosine monophosphate (cAMP) elevating effect. Osthole treatment promoted osteogenesis in osteoblasts by enhancing alkaline phosphatase (ALP) activity and mineralization. Oral gavage of osthole enhanced fracture repair and increased bone strength. Mechanistic study showed osthole triggered the cAMP/CREB pathway through the elevation of the intracellular cAMP level and activation of the phosphorylation of the cAMP response element-binding protein (CREB). Blockage of cAMP/CREB downstream signals with protein kinase A (PKA) inhibitor KT5720 partially suppressed osthole-mediated osteogenesis by inhibiting the elevation of transcription factor, osterix. In conclusion, osthole shows osteopromotive effect on osteoblasts in vitro and in vivo. Osthole-mediated osteogenesis is related to activation of the cAMP/CREB signaling pathway and downstream osterix expression.

## 1. Introduction

Osteoporosis is a systemic skeletal disease characterized by impairment of bone mineral density, strength, and microstructure, leading to an increased risk of fragility fracture that can cause substantial morbidity and mortality [1]. It has become a major public health problem worldwide along with the aging of the population [2]. Osteoporotic fracture treatment has particular difficulties with high rates of implant fixation failure and increased non-union risk caused by delayed bone formation [3,4,5]. Apart from novel technology for enhancement of bone healing [6,7,8], healthy dietary strategies are important modifiable factors for both prevention of osteoporosis and improvement of bone healing [9,10,11]. It is widely accepted that adequate intake of minerals, proteins, and antioxidants enriched foods benefit bone health [12]. Moreover, numerous bioactive phytochemicals found in functional and medicinal food are suggested to have anti-osteoporotic properties [13,14,15,16].

Osthole, a naturally-derived coumarin, is the major bioactive component found in the medicinal herbs *Cnidium monnieri* (L.) Cusson and *Angelica pubescens* Maxim. f., which are commonly used as ingredients in functional foods and herbal medicine formulae [17,18]. Osthole was reported to exert anti-osteoporotic effects in ovariectomy-induced bone loss [19,20]. Moreover, osthole showed in vivo osteoanabolic action by promoting new bone formation in calvaria and endochondral ossification in mice fracture healing [20,21]. Current therapeutic agents of osteoporosis are mainly antiresorptive drugs that inhibit bone resorptive function of osteoclasts, including bisphosphonates, calcitonin, and estrogen analogues. Their efficacy on growth and recovery of bone mass is regarded to be limited [22,23]. On the other hand, bioactive agents that induce osteoblastic bone formation and facilitate fracture repair are suggested more effective and desirable for osteoporosis therapy [24]. Therefore, osthole is a promising potential anabolic agent for osteoporosis and fragility fracture treatment.

The mechanism of the anti-osteoporotic effect of osthole was mainly studied with cell culture models. Previous studies suggested osthole induced osteoblastic differentiation through the bone morphogenetic proteins (BMP)-dependent pathways, which were likely triggered by β-catenin signaling [20,25,26]. More detailed molecular studies are required to enrich the knowledge and to clarify the mechanism of the osteogenic and anti-osteoporotic properties before potential clinical application. On the other hand, multiple studies have demonstrated that cAMP/CREB signaling is the dominant mechanism for the anabolic action of parathyroid hormone (PTH) on bone formation [27,28,29]. In osteoblastic cells and bone tissues, PTH binds to PTH receptors and produces cyclic adenosine monophosphate (cAMP) from adenosine triphosphate (ATP). It leads to the activation of protein kinase A (PKA) and phosphorylation of the cAMP response element-binding protein (CREB) [27,30]. Activated CREB binds to the cAMP response element (CRE) and triggers the cascade expression of osteogenic-related genes [31,32]. Osthole was reported to elevate cAMP levels in adrenocortical cancer cells, trachea, and corpus cavernosum tissues [33,34,35]. Although none reported the cAMP-elevating action of osthole in bone cells or osseous tissues, it was highly probable that osthole treatment would increase cellular cAMP levels in osteoblast cultures and regenerating bone. This might be an alternative possible mechanism of the osteopromotive effect of osthole.

Considering the prospects of osthole as an anabolic anti-osteoporotic agent, the objectives of the present study were to investigate the osteopromotive action of osthole in osteoblasts and bone regeneration during fracture repair. We hypothesized that osthole treatment enhanced osteogenesis in osteoblasts and thereby improved fracture repair by increasing bone strength via cAMP-mediated signaling.

## 2. Materials and Methods

### 2.1. Chemicals and Cell Culture

Osthole (≥95% high performance liquid chromatography, HPLC grade), PKA inhibitor KT5720, and dimethyl sulfoxide (DMSO) were purchased from Sigma-Aldrich Corporation (St. Louis, MO, USA). Recombinant mouse noggin was purchased from R and D Systems, Inc. (Minneapolis, MN, USA). Vehicle control cells were supplied with solvent DMSO (<0.1%). Cell cultures were pre-treated alone with noggin (100 ng/mL) or KT5720 (4 μM) for 1 h and then co-treated with osthole for pathway blockage. All other reagents were obtained from Sigma-Aldrich unless otherwise indicated.

Mouse preosteoblast cell MC3T3-E1 subclone 14 (ATCC CRL-2594) was purchased from the American Type Culture Collection (Manassas, VA, USA). Cells were routinely maintained in complete growth medium containing alpha minimum essential medium (α-MEM) (Thermo Fisher Scientific, Waltham, MA, USA) supplemented with 10% fetal bovine serum (FBS) (Thermo Fisher Scientific) and 1% penicillin-streptomycin-neomycin antibiotic mixture (Thermo Fisher Scientific), incubated at 37 °C in a humidified 5% CO_2_ atmosphere. Cells were differentiated in osteogenic medium supplied with 0.25 mM l-ascorbic acid (l-AA) and 10 mM β-glycerolphosphate (β-GP) in growth medium once reaching 90% confluency. Both growth and differentiation media were renewed every 2 days.

### 2.2. Mouse Femoral Osteotomy

C57BL/6 mice were obtained from the Laboratory Animal Services Center of the Chinese University of Hong Kong. The animal study was approved by the Animal Experimentation Ethics Committee (Ref. No. 13/010/GRF). All mice were first acclimatized and housed at the research animal laboratory during the experimental period. Open osteotomy at the femur shaft was performed on 12-week-old male C57BL/6 mice. Briefly, mice (weight around 25 to 30 grams) were anaesthetized generally with intraperitoneal injection of ketamine (67 mg/kg) and xylazine (13 mg/kg). After shaving and sterilization of the right leg, a lateral incision was made and a 25-gauge needle was inserted retrograde into the intramedullary canal from knee articular surface for internal fixation. Diaphysis of the femur was exposed and an air-driven oscillating saw (Synthes Holding AG, Zuchwil, Switzerland) was used to create a transverse midshaft fracture under irrigation with sterile 0.9% saline solution. Absorbable sutures were used to close the intramuscular septum and skin incision. Osthole reagent was freshly prepared by dissolution with 0.5% (*v*/*v*) Tween 80 in distilled water. Mice were administrated with 20 mg/kg osthole (dosage derived from previous report [19]) in the osthole treatment group (Ost) and vehicle solvent in the control group (Ctl) through daily oral gavage from post-operative day 7 until euthanasia.

### 2.3. Cell Viability/Proliferation Assay

Cells were seeded at a density of 1 × 10^4^ per well into a 96-well plate. After 24 h pre-incubation, they were exposed to osthole at concentrations of 0, 10, 20, 50, 100, 200, and 500 μM for 24, 48, and 72 h. At the end of the treatment, cells were washed with phosphate-buffered saline (PBS) and incubated with 0.5 mg/mL 3-(4,5-cimethylthiazol-2-yl)-2,5-diphenyl tetrazolium bromide (MTT) in medium for an additional 3 h. After incubation, 100 μL DMSO was added each well to dissolve the formazan crystals and the optical density at 570 nm (OD_570_) was measured with a microplate reader (BioTek, Winooski, VT, USA). Cell viability was expressed as the percentage of the control by comparing the viability in the treatment groups to that of the vehicle control group.

### 2.4. Alkaline Phosphatase (ALP) Enzyme-Cytochemistry and ALP Activity Assay

Cells (4 × 10^4^ per well) were seeded into a 24-well plate and allowed to incubate for 48 h in growth medium, and then exposed to 0–100 μM of osthole in osteogenic medium or growth medium for 12 days. After treatment, cells were washed with PBS and fixed with 70% ethanol. Cells were then equilibrated with ALP buffer (50 mM Tris-HCl, pH 9.5, 100 mM NaCl, 50 mM MgCl_2_, 0.1% Tween 20) for 10 min, and incubated with ALP staining solution (5 μL 5-bromo-4-chloro-3-indolyl phosphate (BCIP) and 10 μL nitro blue tetrazolium (NBT) (Promega, Fitchburg, WI, USA) in 1 mL of ALP buffer) in dark at 37 °C. Intensity of ALP staining was assessed by both macroscopic and microscopic observations.

Differentiated and control cells in a 24-well plate were washed with PBS and lysed with 150 μL ice cold lysis buffer (50 mM Tris-HCl, pH 7.4, 100 mM NaCl, 2 mM MgCl_2_, and 0.5% Triton X-100). ALP activities of cell lysate were measured with an ALP-AMP kit (BioSystems Reagents and Instruments, Quezon City, Philippines). Briefly, 50 μL cell lysate was transferred to a 96-well plate, and 150 μL of freshly-prepared substrate working reagent was aliquoted to each well. OD_405_ of the mixture was measured every minute with a microplate reader for 20 min at room temperature. The curve was plotted using absorbance against time, and ΔOD_405_/min obtained was used to calculate enzyme activity. ALP activity was normalized by protein concentration which was measured with a BCA Protein Assay Kit (Thermo Fisher Scientific) at OD_562_.

### 2.5. Calcium Nodule Staining and Quantification

Differentiated and control cells in the 24-well plate were washed with PBS and fixed with ethanol. Cells were stained with 1% alizarin red S (ARS) (pH 4.1) at 37 °C for 30 min. Calcium nodules were visualized by staining with ARS to form an ARS-calcium complex in a chelation process. After washing and removal of free dyes, mineral deposition in the cell culture was assessed and compared by macroscopic and microscopic observation. Quantification of calcium deposits was conducted using an in vitro osteogenesis assay kit (Merck Millipore, Darmstadt, Germany). Briefly, 400 μL of 10% acetic acid was added to each well to extract dyes. Cells and acetic acid were transferred to microcentrifuge tubes and heated to 85 °C for 10 min. Centrifuged supernatant was collected and neutralized with 10% ammonium hydroxide. Samples were then aliquoted to a 96-well plate and measured at OD_405_ along with standards. The linear curve was plotted with ARS concentration against the absorbance of standards, and the ARS concentrations of samples were calculated.

### 2.6. RNA Extraction and Real-Time RT-PCR

Cells (2 × 10^5^ per well) were seeded into six-well plates and allowed to incubate for 48 h. Cells were exposed to 0–50 μM osthole with or without inhibitors for 12 h or 6 days. Total RNA was extracted from cells with TRIzol reagent (Ambion, Foster City, CA, USA). One microgram of RNA was reverse-transcribed into cDNA using oligo (dT) primers and reverse transcriptase (Promega). Quantitative real-time PCR was performed using a QuantiFast SYBR Green PCR Kit (Qiagen, Hilden, Germany), in a total volume of 20 μL containing 1 μL of reverse-transcription product in the presence of a ribonuclease inhibitor (Takara, Kyoto, Japan) and 0.5 μM of sense and antisense primers of target genes, as below (Tech Dragon Limited, Hong Kong, China). BMP-2 forward: 5′-GCTCCACAAACGAGAAAAGC-3′, reverse: 5′-AGCAAGGGGAAAAGGACACT-3′; FGF-2 forward: 5′-ACACGTCAAACTACAACTCCA-3′, reverse: 5′-TCAGCTCTTAGCAGACATTGG-3′; IGF-1 forward: 5′-GGACCAGAGACCCTTTGCGGGG-3′, reverse: 5′-GGCTGCTTTTGTAGGCTTCAGTGG-3′; runt-related transcription factor 2 (Runx2) forward: 5′-AAGTGCGGTGCAAACTTTCT-3′, reverse 5′-TCTCGGTGGCTGGTAGTGA-3′; Osterix (Osx) forward: 5′-ACTGGCTAGGTGGTGGTCAG-3′, reverse: 5′-GGTAGGGAGCTGGGTTAAGG-3′; ALP forward: 5′-AACCCAGACACAAGCATTCC-3′, reverse: 5′-GAGAGCGAAGGGTCAGTCAG-3; osteocalcin (OCN) forward: 5′-CCGGGAGCAGTGTGAGCTTA-3′, reverse: 5′-TAGATGCGTTTGTAGGCGGTC-3; collagen type I (Col-1) forward: 5′-AGAGCATGACCGATGGATTC-3′, reverse: 5′-CCTTCTTGAGGTTGCCAGTC-3′; glyceraldehyde-3-phosphate dehydrogenase (GAPDH) forward: 5′-ACCCAGAAGACTGTGGATGG-3′, reverse: 5′-CACATTGGGGGTAGGAACAC-3′. PCR conditions consisted of a 10 min hot start at 95 °C followed by 45 cycles of 15 s at 95 °C and 30 s at 60 °C. The expression levels of the mRNAs were normalized by GADPH levels and compared to the vehicle control.

### 2.7. Western Blot

Cells were seeded in six-well plates and exposed to 0–50 μM of osthole with or without KT5720 for 6 h. After treatment, cells were lysed in Pierce RIPA lysis buffer (Thermo Fisher Scientific) supplemented with Pierce protease and phosphatase inhibitor mini-tablets (Thermo Fisher Scientific). Equal 30 μg amounts of proteins from the lysate were separated by 10% sodium dodecyl sulfate-polyacrylamide gel electrophoresis (SDS-PAGE) and electrotransferred onto Immun-Blot PVDF membrane (Bio-Rad Laboratories, Hercules, CA, USA). The membrane was blocked with 3% BSA in PBS supplemented with 0.05% Tween-20, followed by overnight incubation at 4 °C with a diluted solution of primary antibody purchased from Cell Signaling (Danvers, MA, USA) against phospho-CREB (p-CREB), CREB, or α-tubulin (loading control). This was followed by incubation with horseradish peroxidase (HRP)-conjugated secondary antibody (Santa Cruz Biotechnology, Dallas, TX, USA) for 1 h at room temperature. The blots were assessed by their enhanced chemiluminescence (ECL) signal using the Pierce ECL Western blotting substrate (Thermo Fisher Scientific). Developed images were captured with Gel Doc molecular imager and ChemiDoc system and analyzed with Image Lab 3.0 software (Bio-Rad).

### 2.8. cAMP Assay

Intracellular cAMP concentration was quantified with cyclic AMP EIA kit (Cayman Chemical, Ann Arbor, MI, USA) based on the competition between labeled cAMP and non-labeled free cAMP in samples. Briefly, cells were incubated in complete growth medium containing 0–100 μM osthole for 2 h. After drug exposure, cells were lysed in 0.1 M HCl for 20 min with agitation, and the supernatants were collected by centrifuge. 50 μL of samples and standards were added into a 96-well plate followed by incubation with cAMP acetylcholine esterase tracer and cAMP EIA antiserum for 18 h at 4 °C. Each sample was developed with addition of Ellman’s reagent and the plate was read at OD_410_ by microplate reader. cAMP concentration was calculated according to the cAMP standard plots and compared to the vehicle control after being normalized with total protein levels.

### 2.9. Histomorphometry and Immunohistochemistry

Femur specimens were harvested at day 14, and fixed and decalcified with calcium chelating solution (0.5 M EDTA/NaOH, pH 7.5) for two weeks. Decalcified bones were then dehydrated and embedded in paraffin wax using a Leica EG Embedding Center (Leica Microsystem, Wetzlar, Germany). Paraffin blocks were sectioned into 5 μm slices and mounted on glass slides. The sections were deparaffinized and stained with safranin O, fast green, and hematoxylin. The histomorphometry analysis was adapted from our previous study [7]. The bone and cartilage area was measured by a blinded observer using Zen 2012 (Zeiss, Oberkochen, Germany). For immunohistochemistry, deparaffinized sections were rehydrated in PBS and treated with 3% hydrogen perioxide in methanol to quench endogenous peroxidases. Antigen retrieval was performed by incubation in 95 °C 10 mM citrate buffer at pH 6.0 for 10 min. After nonspecific binding blocked with UltraVision protein block (Thermo Fisher Scientific), sections was incubated overnight at 4 °C with diluted solution of primary antibodies against Osx and p-CREB (Abcam, Cambridge, UK) and the solution without antibody as a negative control. The sections were then incubated with secondary antibody for 30 min at room temperature. A colorimetric signal was developed with a Liquid DAB+ Substrate Chromogen System (Dako, CA, USA), counter-stained with hemotoxylin, and subjected to blind evaluation.

### 2.10. Bone Biomechanical Test

The strength of the fractured and contralateral femur midshaft was measured by a three-point bending test using a mechanical testing machine (Biomomentum Inc., Laval, QC, Canada) [36]. Briefly, the surrounding soft tissue of femur was removed and then kept moisture with PBS-soaked gauze before testing. The span distance between the two end supports was fixed at 7.35 mm, load was applied on the bone femur midshaft with a displacement rate of 5 mm/min, and the ultimate load for each sample was measured.

### 2.11. Statistical Analysis

Statistical analysis was conducted using GraphPad Prism 5.0 (GraphPad Software, San Diego, CA, USA) and Excel (Microsoft, San Francisco, CA, USA). Mean and standard deviation values (mean ± SD) were calculated for all statistically analyzed parameters. The differences between groups were analyzed using analysis of variance (ANOVA) followed by Turkey’s post-hoc test or unpaired Student’s *t*-tests. The *p*-value less than 0.05 were considered statistically significant.

## 3. Results

### 3.1. Osthole Promoted Osteogenesis in Osteoblasts

**Figure 1:**
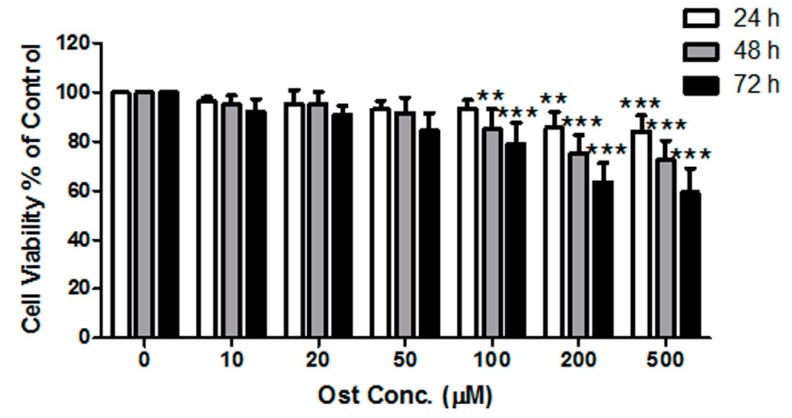
Effect of osthole on proliferation of MC3T3-E1 cells. Cells were treated with 0–500 μM of osthole for 24, 48, and 72 h. The viability was monitored with MTT assay. *n* = 6, One-way ANOVA followed by Tukey’s test and compared to the vehicle control, ** *p* < 0.01, *** *p* < 0.001.

**Figure 2:**
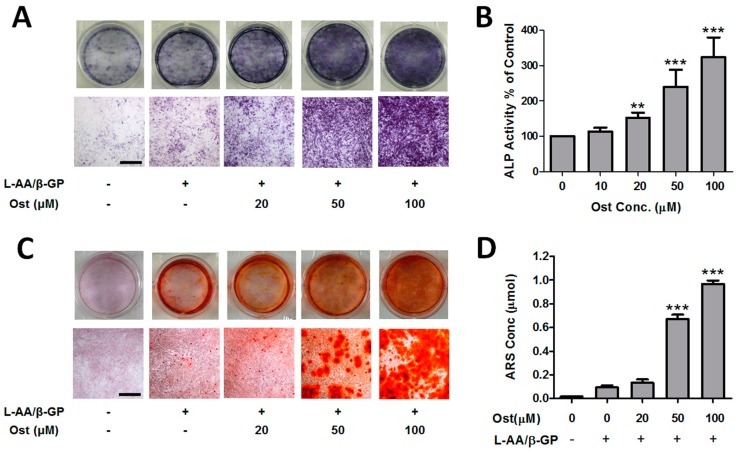
Osthole promoted osteogenic differentiation in osteoblasts. MC3T3-E1 cells were treated with 0–100 μM osthole in growth or osteogenic medium for 12 (**A**,**B**) or 24 days (**C**,**D**). (**A**) Representative macroscopic and microscopic photos of ALP staining cells (*n* = 4); (**B**) ALP activities were measured by ALP-AMP kit (*n* = 6); (**C**) Representative photos of ARS staining cells (*n* = 4); (**D**) cell mineralization was quantified by extraction of ARS dye (*n* = 4). One-way ANOVA was followed by Tukey’s test and compared to the vehicle control, ** *p* < 0.01, *** *p* < 0.001. Bar = 500 μm.

MTT results demonstrated that osthole inhibited the proliferation of MC3T3-E1 cells in a time- and concentration-dependent manner from 0 to 500 μM (Figure 1). Osthole did not lead to significant inhibition on cell proliferation at concentrations less than 100 μM. Thus, 100 μM or below of osthole would be applied on the osteoblastic differentiation in the following experiments. Osteoblastic differentiation was assessed with two measurements: ALP activity and calcium nodule formation. The ALP enzyme cyotochemistry showed that the ALP-positive cell colony number increased with osthole concentration (Figure 2A). ALP activity was also significantly increased by osthole in the range of 0–100 μM in a dose-dependent manner (Figure 2B). In ARS staining of calcium nodule formation, the number and area of stained nodules increased dramatically in osthole-treated cells at concentrations of 50 and 100 μM (Figure 2C) compared with the control and low concentration of osthole. Quantitative analysis of ARS concentration was consistent with the qualitative results (Figure 2D).

### 3.2. Osthole Promoted Bone Regeneration and Bone Strength

**Figure 3:**
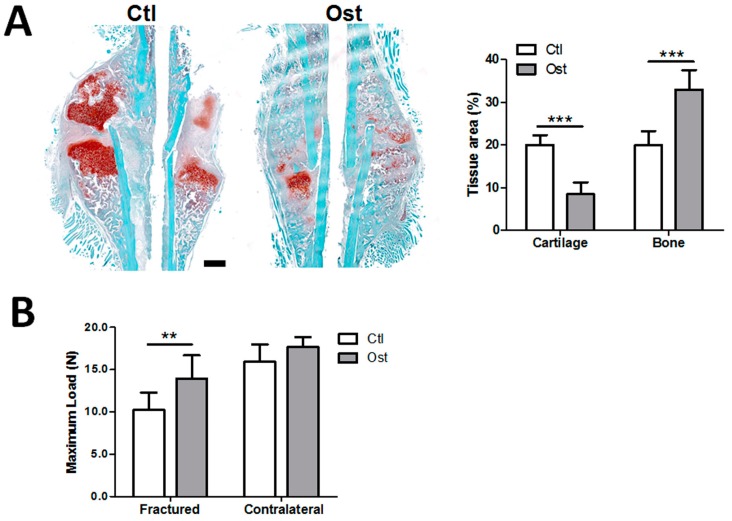
Osthole enhanced bone growth and bone strength during fracture repair. Post-operated fractured and contralateral bones were harvested at day 14 and day 28. (**A**) Callus sections at day 14 were stained with safranin O (cartilage)/fast green and hematoxylin (*n* = 4). Osthole treatment reduced cartilaginous tissue area, but increased bone tissue area; (**B**) the maximum load of femur samples at day 28 was determined by three-point bending (*n* = 8). An unpaired Student *t*-test was compared between control and osthole group, ** *p* < 0.01, *** *p* < 0.001. Bar = 500 μm.

To determine the effect of osthole administration on bone regeneration process in vivo, mice were treated with osthole and vehicle solvent during the fracture repair period. The bone volume of healing fractured callus at day 14 was measured by histomorphometric analysis, and strength of the fractured and contralateral bones was measured at day 28 by a three-point bending test. As shown in Figure 3A, the longitudinal section of calluses in the Ost group contained more bony tissue area and less cartilaginous tissue area than those in Ctl group. Histomorphometric analysis also confirmed the bony callus area in the Ctl group was significantly smaller than Ost group (19.95 ± 3.30 vs. 32.98 ± 4.60); while the cartilaginous callus area was much larger (20.02 ± 2.30 vs. 8.50 ± 2.77), indicating faster growth of bone in the treatment group. Additionally, the maximum load of healing fractured femurs in Ost group (13.96 N) were significantly higher than that in the Ctl group (10.23 N). The maximum load of the contralateral femur in the Ost group was also slightly higher than the Ctl group by 10.76%, but without significant difference (Figure 3B).

### 3.3. Osthole Induced Osteogenesis via the BMP-Dependent Signaling Pathway

**Figure 4:**
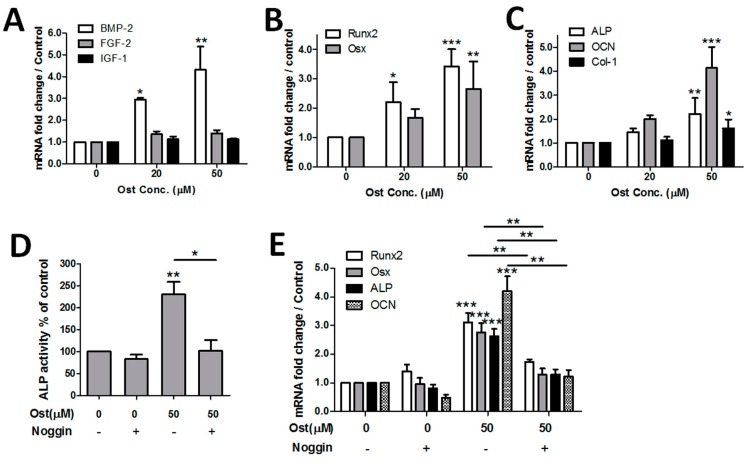
Osthole activated the BMP-2-dependent signaling pathway and BMP antagonist noggin completely inhibited osthole-mediated osteogenesis. MC3T3-E1 cells were treated with 0–50 μM osthole in osteogenic medium in the presence or absence of 100 ng/mL noggin for 12 h (**A**), 6 days (**B**,**C**,**E**), or 12 days (**D**). ALP activity was measured by an ALP-AMP kit; gene expression levels were detected by real-time RT-PCR. (**A**) *n* = 5, (**B**–**E**) *n* = 4; one-way ANOVA, followed by Tukey’s test, was compared to the vehicle control; unpaired Student *t*-test was compared between noggin ±, * *p* < 0.05, ** *p* < 0.01, *** *p* < 0.001.

Expression of osteogenic-related genes was measured in osthole-treated MC3T3-cells to explore the mechanism of the osteopromotive action. The mRNA levels of growth factors (BMP-2, FGF-2 and IGF-1) were measured at 12 h while transcription factors (Runx2 and Osx) and osteogenic maker genes (ALP, OCN and Col-1) were measured at 6 days. Results showed that BMP-2 expression was upregulated in a dose-dependent manner under 0–50 μM of osthole exposure. In contrast, mRNA levels of FGF-2 and IGF-1 did not change significantly (Figure 4A). Both transcription factor genes were upregulated dose-dependently by osthole (Figure 4B). Osthole also significantly activated downstream ALP, OCN, and Col-1, whereas the effect on Col-1 was relatively weak (Figure 4C). To examine the role of BMP signaling in osthole-induced osteogenesis, BMP antagonist noggin was applied to block the BMP-dependent pathway. Osteogenic marker ALP activity, calcium deposits, and osteogenic-related genes were quantified with or without noggin. Application of noggin completely inhibited osthole-induced ALP activation, without a notable change of ALP in vehicle control (Figure 4D). The expression level of osteogenic-related genes Runx2, ALP, and OCN evoked by osthole were also significantly downregulated by co-treatment with noggin (Figure 4E).

### 3.4. Osthole Activated cAMP/CREB Signaling Pathway

**Figure 5:**
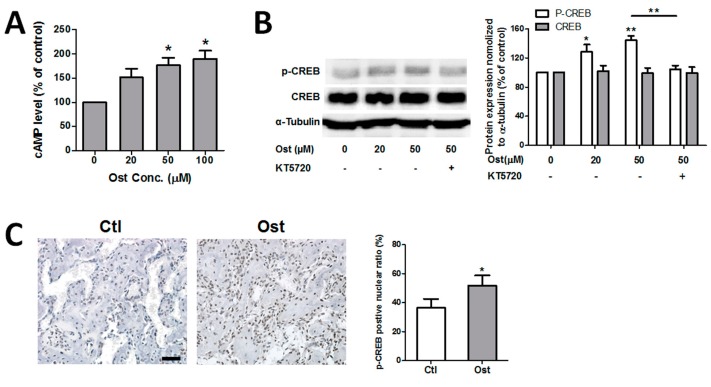
Osthole activated cAMP/CREB pathway. (**A**) MC3T3-E1 cells were incubated with 0–100 μM osthole in growth medium for 2 h, and cellular cAMP levels were quantified with cAMP EIA kit (*n* = 3); (**B**) Cells were treated with 0, 20, or 50 μM osthole in osteogenic medium in the presence or absence of 4 μM KT5720 for 6 h. Proteins were separated by 10% SDS-PAGE and assessed with Western blotting, and the intensity of the bands was quantified (*n* = 5); (**C**) Callus sections at day 14 were blotted with p-CREB antibody and counterstained with hematoxylin. The p-CREB-positive nuclei percentage was counted (*n* = 4). One-way ANOVA, followed by Tukey’s test, was compared to the vehicle control; an unpaired Student *t*-test was compared between KT5720 ± groups, * *p* < 0.05, ** *p* < 0.01. Bar = 50 μm.

To testify whether osthole triggered the cAMP/CREB pathway or not, the effect of osthole on cellular cAMP concentration and CREB phosphorylation were evaluated in MC3T3-E1 cells. Quantification of cAMP demonstrated that osthole significantly elevated the intracellular cAMP level dose-dependently from 0 to 100 μM (Figure 5A). In addition, osthole exposure activated phosphorylation of CREB significantly, but not in the total amount of CREB (Figure 5B). Both results suggested that osthole treatment activated cAMP/CREB signaling in osteoblasts. Immunohistochemistry of phosphorylated CREB (p-CREB) was compared between osthole-treated fractured femurs and control. The p-CREB was only found in nuclei. The percentage of p-CREB positive cells was calculated by the average value of six random views in the bony callus. After treatment with osthole, the percentage of p-CREB-positive nuclei in newly-formed bone significantly increased and the signal intensity in the nuclei was also notably enhanced (Figure 5C).

### 3.5. Osthole Enhanced Osteogenesis through Osterix Activiated by cAMP/CREB Signaling

**Figure 6:**
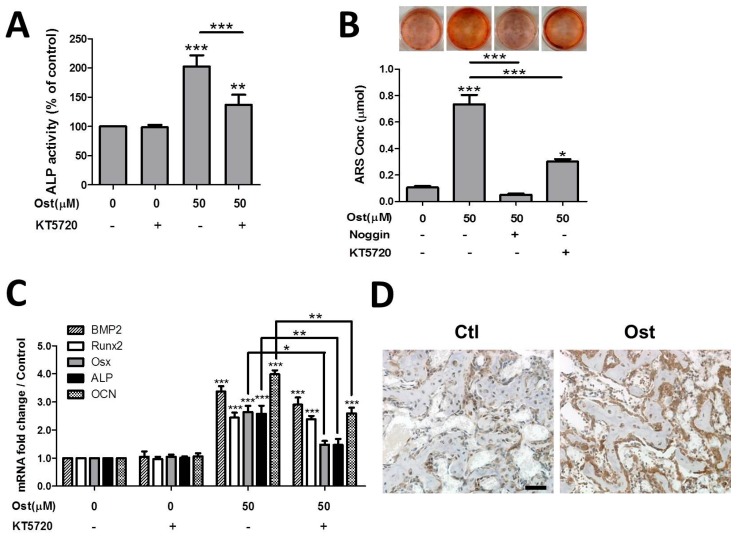
PKA inhibitor partially suppressed osthole-evoked osteogenesis and Osx expression. MC3T3-E1 cells were treated with 0 or 50 μM osthole in osteogenic medium in the presence or absence of 4 μM KT5720 or 100 ng/mL noggin for 12 days (**A**), 24 days (**B**) 12 h or 6 days (**C**). (**A**) ALP activity was measured by ALP-AMP kit (*n* = 5), (**B**) calcium nodules were stained with ARS, and cell mineralization was quantified by extraction of ARS dye (*n* = 3); (**C**) gene expression levels were detected by real-time RT-PCR (*n* = 4); (**D**) callus sections at day 14 were blotted with osterix antibody and counterstained with hematoxylin (*n* = 4). One-way ANOVA was followed by Tukey’s test compared to the vehicle control; unpaired Student *t*-test compared between KT5720 ± or noggin ± group, * *p* < 0.05, ** *p* < 0.01, *** *p* < 0.001. Bar = 50 μm.

The effects of osthole-activated cAMP/CREB signaling activity was determined by the application of PKA inhibitor KT5720 during osteoblast differentiation. Co-treatment of KT5720 with osthole completely blocked the activation of p-CREB in osthole-mediated cAMP/PKA signaling (Figure 5B). PKA inhibitor markedly suppressed both ALP activation and mineralization mediated by osthole; whereas these two osteogenic markers were still significantly higher than the vehicle control after complete blockage of osthole-induced PKA signaling (Figure 6A,B). Additionally, KT5720 exposure alone did not change the viability or differentiation status of cells. All results suggested that PKA blockage only partially suppressed the osteogenic differentiation activated by osthole. Osteogenic-related gene expressions were measured in the presence or absence of KT5720. Consistent to the results of ALP activity and calcium deposit, ALP and OCN gene expression were evidently downregulated by PKA inhibitor, but they were still higher than control levels by 0.47 and 1.59 folds, respectively (Figure 6C). Expression level of Osx was markedly reduced by PKA inhibition, but BMP-2 and Runx2 levels remained almost unchanged (Figure 6C). Likewise, KT5720 had no significant influence on expression levels of osteogenic-related genes when applied alone. On the other hand, expression of Osx in healing fractured bone was assessed by IHC. The expression of Osx in the Ost group was observably higher than that of the Ctl group in osteogenic cells surrounding newly-formed bone tissue (Figure 6D).

## 4. Discussion

Mouse calvarial preosteoblast MC3T3-E1 with a similar cellular response to primary calvarial osteoblasts was used in this study. Previous studies reported that osthole stimulated growth of osteoblast-like UMR106 cells and primary rat osteoblasts [37,38]. However, others found that osthole only stimulated osteoblastic differentiation at the same doses but not cell proliferation [20,25]. Our results showed that 0–50 μM osthole exhibited little effect on cell proliferation at all time points, whereas 100 μM showed slightly inhibited cell growth at 48 h without notable cell death morphology. This was reasonable since temporal arrest in the G1 phase of the cell cycle was regarded as a prerequisite for cell differentiation, and osthole exposure mediated osteogenic differentiation in osteoblasts [39,40]. Additionally, results showed that the mitogenic growth factor FGF-2 level only slightly increased without significant difference and the IGF-1 level remained almost unchanged after osthole treatment. FGF-2 and IGF-1 were both regarded as mitogens of osteoblastic cell proliferation [41,42], which might explain why there was not any promotive effect of osthole on the proliferation of this cell line.

Increasing in ALP activity and calcium nodule formation in osthole-treated cells suggested that it promoted osteogenic differentiation, which was consistent with previous findings in other osteoblastic-like cells. We also found that osthole treatment not only mediated osteogenesis in osteoblasts in vitro, but also enhanced the ossification process in vivo, which resulted in faster bone regeneration during bone repair. Time point day 14 was located in the reparative phase of fracture repair when the bone grew inside the callus and replaced cartilage until bony fusion. At post-operation day 28, the remodeling phase took place when fractured bone was restored to its original shape and strength after the completed fusion of bony callus [21]. Osthole administration resulted in faster bone formation during repair, which led to stronger bone at the end point after bony fusion. This finding echoed with the osteoanabolic effect found in mouse calvaria [20]. Moreover, worth mentioning was that the average bone strength in contralateral intact femur was also increased. This result was consistent with findings of previous studies [19] suggesting osthole exhibited an anti-osteoporotic property by raising the bone mineral density and bone strength. Stronger healing bone in the Ost group should be an achievement resulting from both a faster bone regeneration rate and a higher bone mineral density [21]. Since the bone formation rate in heathy bone was much slower than in repairing bone, the strength difference between Ost and Ctl groups in contralateral bone was much smaller.

Previous studies suggested that osthole stimulated osteogenesis in osteoblasts through BMP-dependent pathway [20,25]. Our results confirmed the effect of osthole on BMP-2 expression. Expression of BMPs triggers osteogenic signaling cascades driving the whole process of differentiation. Both key transcription factors of osteogenesis, Osx, and Runx2, were evoked by osthole and so did three downstream osteogenic marker genes ALP, OCN, and Col-1. Moreover, the influence of BMP pathway in osthole-mediated osteogenesis was examined by the measurement of ALP activity, mineralization, and marker genes in the presence of BMP antagonist noggin. The osteopromotive effect of osthole was abolished by blockage of the BMP pathway, which indicated osthole-mediated osteogenesis was completely BMP-dependent.

On the other hand, osthole was suggested to elevate the cAMP level by inhibition of cAMP phosphodiesterases (PDEs), which hydrolyzes cAMP into AMP in various types of tissues. This was the first time cAMP elevation action of osthole was confirmed in osteoblastic cell culture, suggesting the action should be non-tissue-specific. Osthole also induced phosphorylation/activation of CREB, downstream target of cAMP/PKA signaling It indicated that osthole triggered the cAMP/PKA/CREB pathway, similar to PTH and some other cAMP activators that promote osteogenesis [29,43]. The effect of osthole on phosphorylation of CREB was also found in neural cells [44], which further revealed cAMP/CREB activation by osthole was not tissue-specific and might contribute to its multiple bioactivities. PKA inhibitor KT5720 could completely block the cAMP-mediated phosphorylation of CREB induced by osthole; meanwhile, the inhibitor only partially suppressed osthole-mediated osteogenesis. In addition, ALP activity, mineralization, and the osteogenic gene level treated with KT5720 were still significantly higher than that of vehicle controls, which suggested that osthole-induced osteogenesis was only partially mediated by the cAMP/CREB pathway. This finding supported previous suggestions that osthole-induced cell differentiation operated by both Smad-dependent and -independent pathways [25]. cAMP/CREB activators, such as PTH and db-cAMP, were found to directly stimulate growth factors (especially BMP-2) and their downstream target genes in mesenchymal stem cells [43]. However, our results showed only a slight reduction of BMP-2 expression without significant difference after PKA blockage, which indicated that osthole-activated cAMP/CREB signaling might promote BMP-dependent osteogenesis signaling through other downstream elements rather than only evoking BMP expression in this cell line.

Furthermore, we also found that osthole-activated cAMP/CREB signaling targeted transcription factor Osx rather than Runx2. Although the interaction between Runx2 and Osx remains controversial, both transcription factors have been found indispensable in both in vitro and in vivo osteogenesis/ossification process [45,46,47]. Previous studies found deficiencies of either Osx or Runx2 genes leading to a complete absence of bone formation at the embryonic stage, but the phenotype of Osx null mice were different from Runx2 null mice at birth [47]. Others reported that Osx and Runx2 regulated distinct gene groups [48]. Both suggested that Osx and Runx2 were critical, but with distinct functions in bone formation progress. Osteogenesis in osteoblasts was mediated through the BMP-dependent pathway while the key transcription factor Osx was further upregulated via cAMP/CREB signaling. Meanwhile, single activation of Osx by cAMP signaling was not sufficient for osteogenic cascade in osteoblasts, probably because it lacked some key elements induced by other transcription factors, such as Runx2. This might be the reason why the blockage of the BMP pathway completely stopped osteogenic differentiation, while inhibition of cAMP/CREB could only partially suppress ALP activity, cell mineralization and osteogenic gene expression. In the mouse bone repair model, we also found that osthole treatment promoted the expression of p-CREB and Osx in osteoblastic cells showing newly-formed woven bone. This indicated that osthole also activated the cAMP/CREB pathway in osteoblasts in vivo. Faster bone formation during repair process might also relate to activation of CREB and Osx in bone forming cells. Thus, apart from the BMP-dependent pathway, osthole also enhanced osteogenesis through cAMP/CREB signaling by upregulating transcriptional factor Osx in osteoblasts.

Several limitations in this study need to be addressed. PKA-specific inhibitor KT5720 was applied for investigation of the influence of osthole-evoked cAMP elevation on osteogenesis. In spite of the wide usage of KT5720 for the inhibition of the cAMP/PKA/CREB pathway, it would be preferable to use RNA interference to specifically knock down the target pathway. Osthole-induced upregulation of p-CREB and Osx in healing bone were insufficient to prove the related mechanism applied in vivo as well. However, these results supported future study of this promising pathway in animal models. Selectively blocking this pathway should be applied to explore the osteoanabolic effect of osthole in vivo.

## 5. Conclusions

In summary, this study had demonstrated that osthole promoted osteogenic differentiation in osteoblasts by the BMP-dependent pathway, which was enhanced by cAMP/CREB signaling targeting the transcription factor osterix. Osthole treatment upregulated osterix expression and promoted bone regeneration during mice femoral fracture repairing. The findings of the study contribute to the knowledge of the mechanism involved in osteopromotive and anti-osteoporotic effects of osthole. It provides biological evidence for diet supplementary of osthole and osthole-contained functional food or medicine for prevention and therapy of osteoporosis and osteoporotic fracture.
